# Based on Network Pharmacology and Molecular Docking, the Active Components, Targets, and Mechanisms of *Flemingia philippinensis* in Improving Inflammation Were Excavated

**DOI:** 10.3390/nu16121850

**Published:** 2024-06-13

**Authors:** Dongying Zhang, Qixing Zhou, Zhen Zhang, Xiangxuan Yang, Jiaxu Man, Dongxue Wang, Xiaoyong Li

**Affiliations:** 1 College of Science, Yunnan Agricultural University, Kunming 650201, China; zhangdongying365@163.com; 2 College of Food and Biological Engineering, Hezhou University, Hezhou 542899, China; 3 College of Food Science and Technology, Yunnan Agricultural University, Kunming 650201, China; zhouqixing0515@163.com (Q.Z.); zhangzhen162897412@163.com (Z.Z.); anxuan200@163.com (X.Y.); 15025171365@163.com (D.W.); 4 Institute of Agricultural Products Processing, Yunnan Academy of Agricultural Sciences, Kunming 650201, China; mjxmjx05@163.com

**Keywords:** *Flemingia philippinensis*, improve inflammation, network pharmacology, molecular docking

## Abstract

*Flemingia philippinensis*, a polyphenol-rich plant, holds potential for improving inflammation, but its mechanisms are not well understood. Therefore, this study employed network pharmacology and molecular docking to explore the mechanism by which *Flemingia philippinensis* ameliorates inflammation. In this study, 29 kinds of active ingredients were obtained via data mining. Five main active components were screened out for improving inflammation, which were flemichin D, naringenin, chrysophanol, genistein and orobol. In total, 52 core targets were identified, including AKT serine/threonine kinase 1 (AKT1), tumor necrosis factor (TNF), B-cell lymphoma-2 (BCL2), serum albumin (ALB), and estrogen receptor 1 (ESR1). Gene ontology (GO) enrichment analysis identified 2331 entries related to biological processes, 98 entries associated with cellular components, and 203 entries linked to molecular functions. Kyoto Encyclopedia of Genes and Genomes (KEGG) enrichment analysis yielded 149 pathways, including those involved in EGFR tyrosine kinase inhibitor resistance, endocrine resistance, and the PI3K–Akt signaling pathway. Molecular docking results showed strong binding effects between the main active components and the core targets, with binding energies less than −5 kcal/mol. In summary, this study preliminarily elucidated the underlying mechanisms by which *Flemingia philippinensis*, through a multi-component, multi-target, and multi-pathway approach, ameliorates inflammation. This provides a theoretical foundation for the subsequent application of *Flemingia philippinensis* in inflammation amelioration.

## 1. Introduction

With the rapid development of society, the research and treatment of inflammatory diseases face new challenges and opportunities. Inflammation is the protective response of organisms to tissue injury or infection, and it is the dynamic defense response of organisms to invading pathogens. This reaction involves a series of complex processes, including phagocytosis, the release of inflammatory cytokines and chemokines, which can help organisms destroy and eliminate pathogens [1]. However, excessive and persistent inflammation can cause irreversible damage to the organism itself, and even endanger life [2]. At the same time, inflammation plays an important role in the occurrence and development of many diseases. For example, the persistent occurrence of inflammatory reaction plays an important role in the process of cancer initiation, promotion, malignant transformation, invasion, and metastasis [3]. In addition, inflammation is also one of the main causes of heart disease, and chronic inflammation can induce an arterial injury process, such as atherosclerosis [4]. At present, some studies have regarded inflammatory regression as a new method to treat inflammatory diseases [5]. At present, the therapeutic effect of anti-inflammatory drugs has been fully confirmed. Dexamethasone, as a glucocorticoid, is mainly used to treat inflammatory diseases [6]. However, long-term use of such drugs usually leads to serious life-threatening side effects [7]. Therefore, it is particularly important to find products with excellent curative effects and little toxicity as anti-inflammatory drug components.

Phenolic metabolites in plants are one of the main sources of substances that ameliorate inflammation [8]. Polyphenols, also known as phenolic compounds, are a class of chemical substances containing aromatic rings and at least two hydroxyl groups. They are widely found in plants and have excellent pharmacological and nutritional properties, including antiviral, antioxidant, and anti-inflammatory activities [9]. *Flemingia philippinensis* (Merr. et Rolfe) is a Chinese herbal medicine rich in polyphenols, especially isoflavone derivatives. This Chinese herbal medicine is planted on a large scale, mainly distributed in Yunnan, Guizhou, Guangxi, and Sichuan, and widely eaten by local residents as an important nutritional health product [10]. As a traditional medicinal and edible plant, *Flemingia philippinensis* from the Philippines has been widely applied in the treatment of various diseases, including chronic nephritis and rheumatoid arthritis. Additionally, it has demonstrated potential therapeutic effects in alleviating pain and improving certain gynecological conditions [11,12,13]. Existing research has indicated that the flavonoids in *Flemingia philippinensis* extract not only have anti-inflammatory effects, but also have a variety of biological activities such as anti-thrombosis, anti-oxidation, liver protection, and cardiovascular diseases [14]. The presence of these compounds provides a broad basis for the pharmacological action of *Flemingia philippinensis*, making it a potential natural drug resource for the treatment of a variety of diseases. In addition, the study of the chemical composition of the *Flemingia philippinensis* has shown that in addition to flavonoids, it also contains coumarin, saponins, volatile oils, and other compounds [15]. The diversity of these compounds further enhances the medicinal value of *Flemingia philippinensis*, and makes it have potential application prospects in the treatment of different diseases.

Although it has been reported that *Flemingia philippinensis* extract has the effect of improving inflammation, at present, the active ingredients, targets, and mechanisms of improving inflammation are still unclear [16]. However, the integration of bioinformatics and network pharmacology provides a practical method for exploring its mechanism of action [17]. Network pharmacology is a new subject developed based on systems biology, pharmacology, and modern computer technology, which can systematically reveal the active components in drugs and predict the relationship between drug components and gene targets [18,19]. Molecular docking can verify the interaction between active compounds and central therapeutic target [20]. Therefore, based on the research idea of the drug–target pathway, this study obtained the active ingredients and their targets for improving inflammation through multi-platform databases, and used Venny 2.1.0 and Cytoscape 3.9.1 software to construct the topological network of the active ingredient improving inflammation. Network pharmacological analysis technology and molecular docking technology were used to explore the active ingredients, targets, and mechanisms of *Flemingia philippinensis* in improving inflammation, in order to provide a theoretical basis for the effect of *Flemingia philippinensis* in improving inflammation.

## 2. Materials and Methods

**Figure 1:**
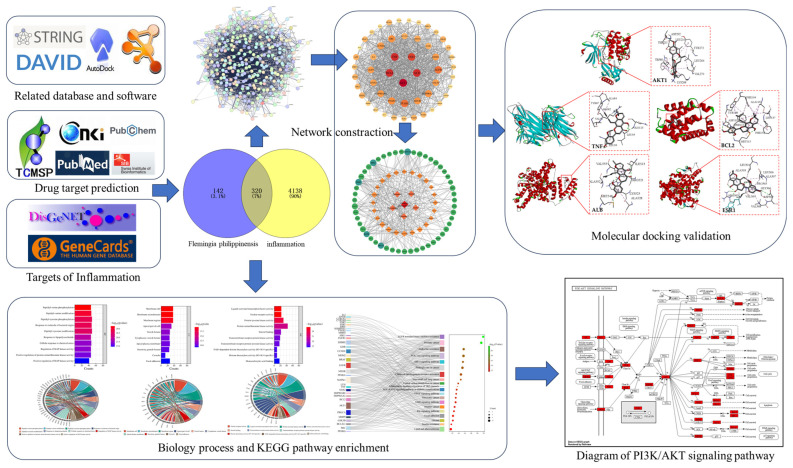
The research flow chart.

Firstly, the main active components were searched in the traditional Chinese medicine systems pharmacology database and analysis platform (TCMSP), the China National Knowledge Infrastructure (CNKI), and the PubMed database, and then combined with a search in the Swiss Target Prediction database, the action targets of each active component were predicted. We obtained zetargets related to inflammation through the Disease Gene Network (DisGENET) and GeneCards databases; Venny 2.1.0 and Cytoscape 3.9.1 were used to construct the *Flemingia philippinensis*–active ingredients–anti-inflammation target network, and the main active components of *Flemingia philippinensis* were screened according to their node degree values. A protein–protein interaction (PPI) network was constructed by the String database, and the key targets of improving inflammation were screened by node degree value. The David database was used for gene ontology (GO) analysis and Kyoto Encyclopedia of Genes and Genomes (KEGG) pathway enrichment analysis. Finally, AutoDock 4.2.6 software was used to perform molecular docking between the main active ingredients and the key targets for improving inflammation (Figure 1).

### 2.1. Searching for the Active Ingredients of Flemingia philippinensis

The active ingredients of *Flemingia philippinensis* were searched through the TCMSP (https://test.tcmsp-e.com/tcmsp.php, accessed on 2 February 2024), CNKI (https://www.cnki.net/, accessed on 2 February 2024), and PubMed (https://pubmed.ncbi.nlm.nih.gov/, accessed on 2 February 2024) databases. Firstly, using TCMSP database, we set “Herb Name” as “*Flemingia philippinensis*” to search and export its active ingredients; secondly, CNKI database and PubMed data were used to search for the active ingredients related to *Flemingia philippinensis*. The repetitive active ingredient obtained via the above steps was removed, that is, the active ingredient of *Flemingia philippinensis* was obtained. We then downloaded the Smile format of these active ingredients from the PubChem (https://pubchem.ncbi.nlm.nih.gov/, accessed on 10 February 2024) database at the time of active ingredient screening, and then from the Swiss ADME (http://www.swissadme.ch/index.php, accessed on 10 February 2024) database, according to the values of ADME screening of the active ingredient. According to Lipinski, Ghose, Veber, Egan, and Muegge, there are at least three ways to dig out the active ingredients of *Flemingia philippinensis*.

### 2.2. Target Prediction for the Active Ingredients of Flemingia philippinensis

The Swiss Target Prediction (http://swisstargetprediction.ch/, accessed on 15 February 2024) database is an online tool for predicting drug targets. It utilizes known compounds with 2D and 3D structure similarity for forecasting compound treatment targets [21]. In the present study, we downloaded the SMILES formats of the active ingredients in *Flemingia philippinensis* from the PubChem database, imported them into the Swiss Target Prediction database, and set the species as “Homo sapiens”. The predictions of the potential targets of the active ingredients were obtained by using “Probability > 0.01” as the screening condition.

### 2.3. Screening for Potential Targets of the Anti-Inflammatory Effects of Flemingia philippinensis

Inflammation-associated targets were obtained from the DisGeNET (https://www.disgenet.org/, accessed on 16 February 2024) and GeneCards (https://www.genecards.org/, accessed on 16 February 2024) databases. “Inflammation” was input into the search box of the DisGeNET database and the GeneCards database to retrieve the related targets. Subsequently, the targets associated with inflammation were obtained, and after deduplication and summarization, the relevant targets were identified. In Section 2.2, the Swiss Target Prediction database was utilized to predict that the active ingredients of *Flemingia philippinensis* potentially target a broad spectrum of diseases. Therefore, with further help from the Venny 2.1.0 (https://bioinfogp.cnb.csic.es/tools/venny/, accessed on 16 February 2024) platform, we screened the intersection of inflammation-related targets with the target of action of the active ingredient of *Flemingia philippinensis*, so as to obtain the potential targets of *Flemingia philippinensis* for ameliorating inflammation.

### 2.4. Analysis of Protein Interaction Network and Screening for Key Targets

The potential targets screened by Section 2.3 were visually analyzed through the String (https://cn.string-db.org/, accessed on 20 February 2024) database. The selected biological species was homo sapiens to obtain the PPI. The obtained PPI network data information was also analyzed with the help of Cytoscape 3.9.1, and topological methods were further used to filter out the core targets in the PPI network graphs based on degree centrality (DC), betweenness centrality (BC), and closeness centrality (CC) [22].

### 2.5. Construction of the Network Diagram of Flemingia philippinensis–Active Ingredients–Targets for Improving Inflammation

The data obtained in Section 2.2 and Section 2.4 were imported into Cytoscape 3.9.1 software, and the network diagram of *Flemingia philippinensis*–active ingredients–targets for improving inflammation was constructed. The larger the node degree value, the darker the node color.

### 2.6. GO Enrichment Analysis and KEGG Pathway Analysis

Through the David (https://david.ncifcrf.gov/, accessed on 25 February 2024) database, GO function analysis and KEGG pathway enrichment analysis were carried out on the potential targets of active ingredients in improving inflammation. The top 10 *p*-values of biological process (BP), molecular function (MF), and cellular component (CC) in the GO analysis were selected for further analysis, including the creation of bar charts and chord charts. Subsequently, the top 20 pathways based on *p*-values were chosen for KEGG pathway enrichment analysis, along with the generation of Sankey bubble diagrams and chord diagrams. Correlations between these pathways were then evaluated. The results of these analyses shed light on the potential mechanisms underlying the anti-inflammatory effects of the active ingredients.

### 2.7. Molecular Docking Analysis

Molecular docking technology can intuitively show the degree of binding between the active components and the target [23]. In order to further verify the interaction between the selected active compounds and the core targets, the top 5 active compounds and the core targets were selected for molecular docking based on the degree values. In this study, AutoDock 4.2.6 was utilized to conduct molecular docking of the main active ingredients, including Flemichin D (CAS: 57096-07-8), Naringenin (CAS: 480-41-1), Chrysophanol (CAS: 481-74-3), genistein (CAS: 446-72-0), and Orobol (CAS: 480-23-9), with the key targets AKT serine/threonine kinase 1 (AKT1) (PDB ID: 7NH5), tumor necrosis factor (TNF) (PDB ID: 2AZ5), B-cell lymphoma-2 (BCL2) (PDB ID: 6GL8), serum albumin (ALB) (PDB ID: 6R7S), and estrogen receptor 1 (ESR1) (PDB ID: 1L2I). First, the main active ingredient was downloaded from the PubChem database (https://pubchem.ncbi.nlm.nih.gov/, accessed on 5 March 2024) in SDF format and with a 3D structure. It was then imported into Chem3D 20.0 for energy and structure optimization, setting the minimum RMS gradient to 0.001. Ligand preprocessing was conducted using AutoDock 4.2.6, which involved the addition of all hydrogens and the designation of the ligand. After inspecting the rotatable bonds, the ligand was exported as a pdbqt file for subsequent molecular docking calculations [24]. After that, the protein structure of the key target was downloaded from the PDB database (https://www.rcsb.org/, accessed on 5 March 2024) and imported into PyMOL 2.5 to remove excess ligands. Then, the structure of the key protein molecule was pretreated with AutoDock 4.2.6, including dehydration and hydrogenation, and used as the receptor. Selected as the receptor, it was then exported as a pdbqt file for subsequent molecular docking calculations [25]. To identify the binding site of the receptor, DoGSiteScorer (https://proteins.plus/, accessed on 5 March 2024) developed by the Bioinformatics Centre at Universität Hamburg was utilized for the online prediction of binding pockets [26]. Finally, AutoDock 4.2.6 was used to perform molecular docking and calculate binding energy, and Discovery Studio 2021 was used to produce graphs.

## 3. Results

### 3.1. Active Ingredients of Flemingia philippinensis

The traditional Chinese medicine systems pharmacology database and analysis platform (TCMSP), China National Knowledge Infrastructure (CNKI), and PubMed databases were used to search for the active ingredients of *Flemingia philippinensis*. After collecting and removing the repeated values and invalid ingredients, 29 active ingredients in total were obtained [16,27,28,29,30,31] (Table 1).

### 3.2. Prediction of the Potential Targets for Anti-Inflammatory Improvement by Flemingia philippinensis

**Figure 2:**
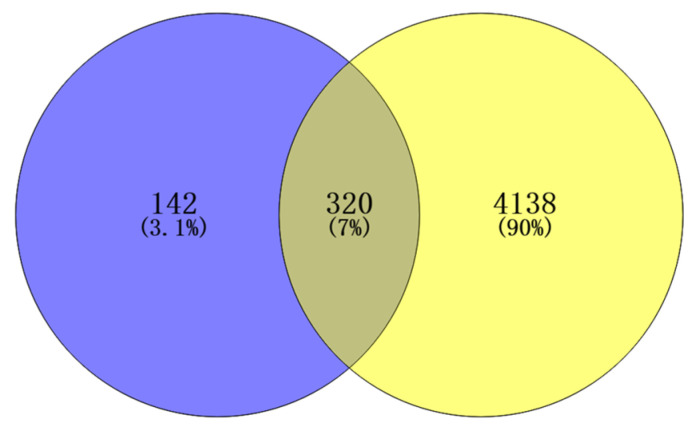
Intersection targets of active ingredients and inflammation in *Flemingia Philippinensis* extract.

An initial search in the Disease Gene Network (DisGeNET) and GeneCards databases identified 4458 inflammation-related targets. All the targets of *Flemingia philippinensis* were predicted by the Swiss Target Prediction database, and 462 were obtained. Potential targets of *Flemingia philippinensis* for ameliorating inflammation were screened using Venny 2.1.0, and 320 in total were obtained (Figure 2).

### 3.3. Protein Interaction Analysis

**Figure 3:**
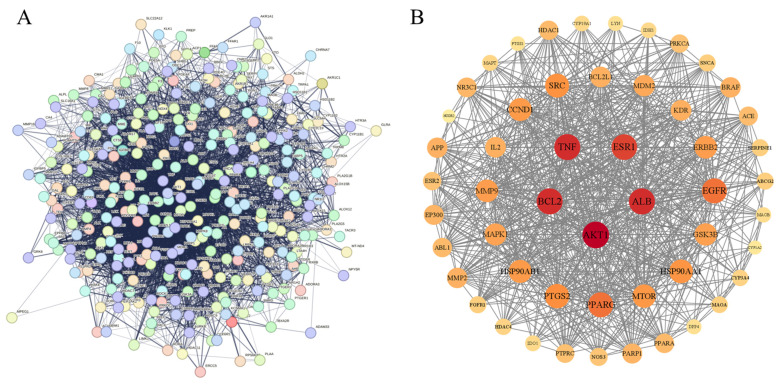
(**A**) PPI network diagram derived from the STRING database; (**B**) diagram of core target construction using Cytoscape software. In Figure (**B**), nodes represent proteins, edges represent connections between proteins, and the size and color of nodes indicate the importance of each node within the entire protein interaction network.

Using the STRING database, protein interaction analysis was carried out on 320 intersection targets for improving inflammation from *Flemingia Philippinensis*, and a protein–protein interaction (PPI) network was constructed (Figure 3A). In total, 319 nodes and 5264 connecting lines between nodes were obtained. In order to further screen the core target proteins in the PPI network, this study used Cytoscape 3.9.1 to analyze the topology of PPI network nodes, and selected the target values that were greater than those of degree centrality (DC), betweenness centrality (BC), and closeness centrality (CC). In total, 52 core targets including protein AKT serine/threonine kinase 1 (AKT1), serum albumin (ALB), B-cell lymphoma-2 (BCL2), tumor necrosis factor (TNF), and estrogen receptor 1 (ESR1) were obtained (Figure 3B, Table 2).

### 3.4. Network of Flemingia philippinensis–Active Ingredients–Their Targets for Inflammation Improvement

**Figure 4:**
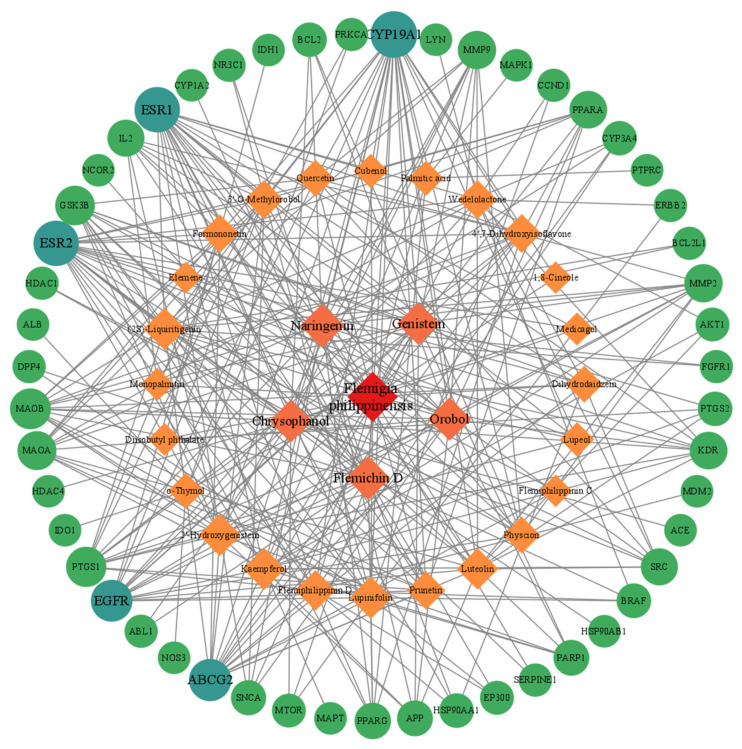
The target network of *Flemingia philippinensis*–active ingredients improving core targets of inflammation. In the diagram, rhomboids represent the active ingredients of *Flemingia philippinensis* that improve inflammation, and circles represent the core targets for inflammation improvement.

The *Flemingia philippinensis*–active ingredient–inflammatory core targets network shows that (Figure 4), in addition to *Flemingia philippinensis*, five ingredients, Flemichin D, Naringenin, Chrysophanol, Genistein, and Orobol, had the darkest nodes, suggesting that they are the main active components of *Flemingia philippinensis* in improving inflammation. In addition, each target node was connected by multiple edges, indicating that the anti-inflammatory effects of *Flemingia philippinensis* result from the synergistic actions of various compounds.

### 3.5. GO Enrichment Analysis of Potential Inflammatory Targets in Flemingia philippinensis

**Figure 5:**
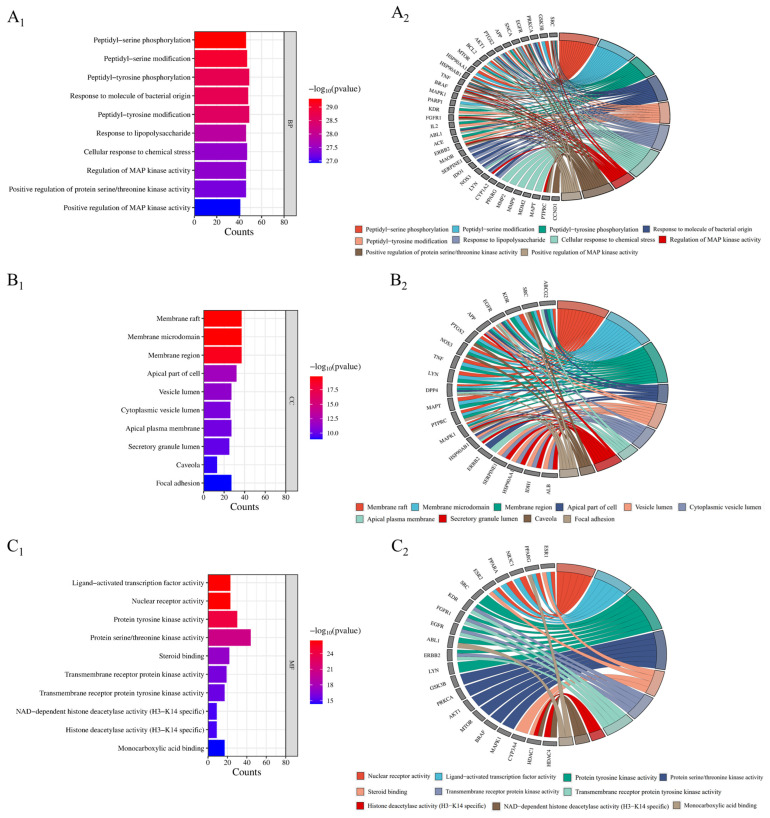
GO functional enrichment analysis (**A_1_**–**C_1_**) and core anti-inflammatory targets (**A_2_**–**C_2_**).

In total, 2632 items were involved in the gene ontology (GO) enrichment analysis of potential targets for improving inflammation. The top 10 items in each section were ranked from the lowest to highest *p*-value (Figure 5). The biological processes included 2331 biological processes such as peptidyl-serine phosphorylation, peptidyl-serine modification, peptidyl-tyrosine phosphorylation, etc. Cell composition included 98 components such as membrane rafts, membrane microdomains, membrane regions, etc. Molecular functions included 203 functions such as nuclear receptor activity, ligand-activated transcription factor activity, protein tyrosine kinase activity, etc. (*p* < 0.01).

### 3.6. KEGG Pathway Enrichment Analysis of the Potential Anti-Inflammatory Targets of Flemingia philippinensis

**Figure 6:**
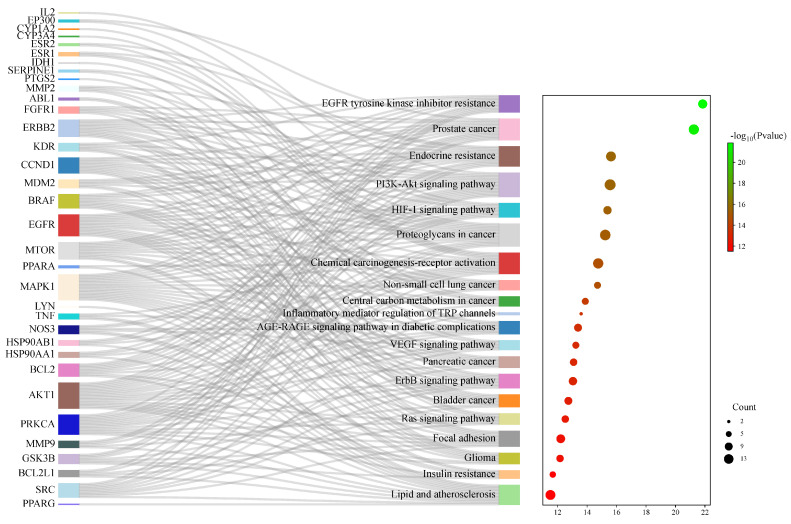
KEGG signaling pathway enrichment analysis of potential targets for anti-inflammatory improvement. The Sankey plot on the left represents the core targets included in each pathway, and the conventional bubble plot is on the right, where the bubble size indicates the number of potential targets to which the pathway belongs, and the bubble color indicates the *p*-value.

**Figure 7:**
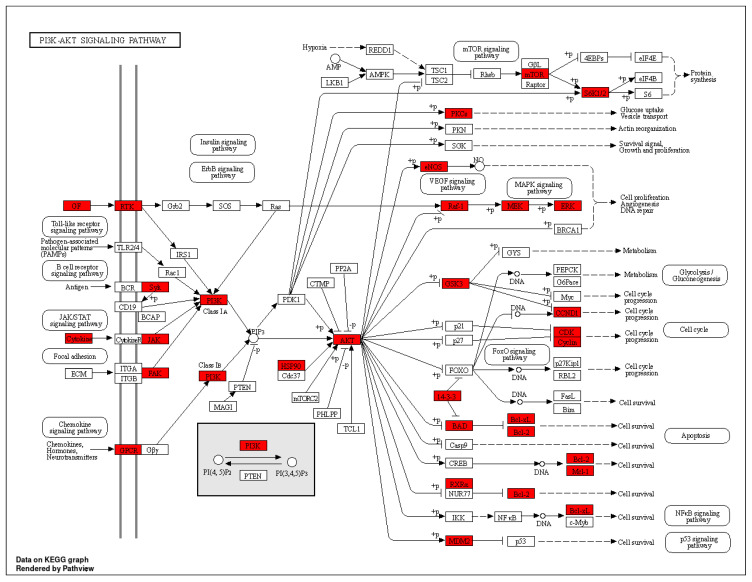
Diagram of PI3K–AKT signaling pathway. Red nodes indicate genes associated with improved inflammation in *Flemingia philippinensis*.

In total, 149 related pathways were obtained via Kyoto Encyclopedia of Genes and Genomes (KEGG) pathway enrichment analysis. (*p* < 0.01). According to the *p*-value, the top 20 pathways were sorted, and a Sankey bubble plot was created (Figure 6). The results showed that the targets of *Flemingia philippinensis* in improving inflammation were mainly involved in EGFR tyrosine kinase inhibitor resistance, prostate cancer, and endocrine resistance. Additionally, the PI3K–Akt signaling pathway and the HIF-1 signaling pathway play important roles in improving inflammation. Among the core targets identified, 12 are involved in resistance to EGFR tyrosine kinase inhibitor resistance, 15 are associated with prostate cancer, 14 are implicated in endocrine resistance, 17 are engaged in the PI3K–Akt signaling pathway, and 10 are involved in the HIF-1 signaling pathway. Among them, the PI3K–Akt pathway, with the most core targets, may be the most important pathway in which *Flemingia philippinensis* improves inflammation (Figure 7). In addition, multiple core targets were found to be involved in multiple pathways simultaneously. When *Flemingia philippinensis* regulates a specific pathway, changes in target expression may have chain or indirect effects on other pathways. The results showed that *Flemingia philippinensis* could improve inflammation by regulating multiple targets and related pathways, and there were high correlations among different pathways.

### 3.7. Analysis of Molecular Docking

**Figure 8:**
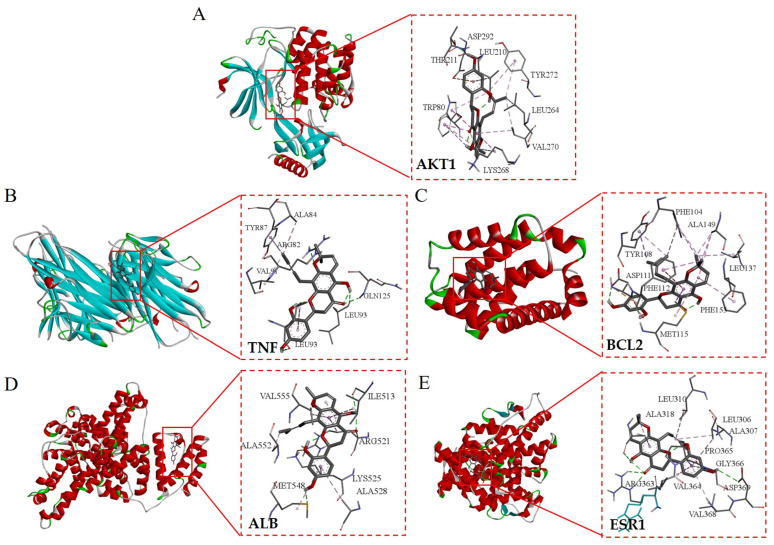
Visualization of molecular docking of the active ingredient in *Flemingia philippinensis* (Flemichin D) with the key target. Figures (**A**–**E**) respectively display the molecular docking outcomes of Flemichin D with the following proteins: AKT1 (Figure **A**), TNF (Figure **B**), BCL2 (Figure **C**), ALB (Figure **D**), and ESR1 (Figure **E**).

The top five core target proteins identified, AKT1, TNF, BCL2, ALB, and ESR1, were subjected to molecular docking analysis with the top five principal active components known for their anti-inflammatory properties, namely Flemichin D, Naringenin, Chrysophanol, Genistein, and Orobol. The results showed that the binding energies between the main active ingredients and the core target proteins were all less than -5 kcal/mol, indicating that the predicted active ingredients had good binding properties with the key targets. (Table 3 and Figure 8). Among them, the binding energy of Flemichin D to the core target protein was lower than that of the other components, indicating that Flemichin D plays an important role in improving inflammation in *Flemingia philippinensis*.

## 4. Discussion

Inflammatory processes are known to play a key role in a variety of psychosomatic pathologies, including ischemic heart disease, diabetes, neurodegenerative diseases, depression, and schizophrenia [32]. Most of these diseases are considered to be chronic inflammatory diseases and exhibit high morbidity and mortality worldwide [33]. Nonsteroidal anti-inflammatory drugs (NSAIDs) are a class of non-glucocorticoid medications widely used to ameliorate inflammation [34]. Although they can provide temporary relief of inflammatory responses, their long-term therapeutic efficacy is limited and cannot cure the disease. Additionally, prolonged use of NSAIDs may lead to gastrointestinal, hepatic, and renal damage, as well as adverse effects on the hematopoietic system and the central nervous system [35]. Plant resources show great potential in the improvement of inflammatory conditions. Numerous plants are rich in natural anti-inflammatory constituents, such as polyphenols, flavonoids, and terpenes. These components significantly alleviate inflammatory responses by inhibiting the release of inflammatory mediators, reducing oxidative stress, and modulating immune responses [36,37,38]. Although the anti-inflammatory effects of *Flemingia philippinensis* have been reported, the underlying mechanisms remain unclear. To further explore the bioactive role of *Flemingia philippinensis* in improving inflammation, this study, for the first time, systematically investigated the mechanisms by which the active components of *Flemingia philippinensis* improve anti-inflammatory effects using a combination of network pharmacology and molecular docking methods. The results showed that *Flemingia philippinensis* had a multi-component and multi-target effect, and different active ingredients exerted their anti-inflammatory effect.

Topological network analysis showed that the nodes of Flemichin D, Naringenin, Chrysophanol, Genistein, and Orobol were the largest, indicating that these five components are the main active components of *Flemingia philippinensis* in improving inflammation. The PPI network analysis revealed strong interaction effects between the active components of *Flemingia philippinensis* and their target proteins. According to the degree values, AKT serine/threonine kinase 1 (AKT1), tumor necrosis factor (TNF), B-cell lymphoma-2 (BCL2), serum albumin (ALB), and estrogen receptor 1 (ESR1) are the top five key targets. Molecular docking results demonstrated that the top five active ingredients could spontaneously bind to the top five target proteins, with Flemichin D being the most significant in improving inflammation. Flemichin D is the major flavonoid in *Flemingia philippinensis*. However, it has not been reported that it can improve inflammation [31]. Related studies have shown that Flemiphilippinin D, a compound similar to Flemichin D, can reduce the secretion of pro-inflammatory factors and reduce the inflammatory response through TLR2/MyD88/NF-κB signaling pathway, thus playing an anti-rheumatic effect [39]. This suggests that Flemichin D is also a potential inflammation-ameliorating compound. Naringenin is a flavonoid widely present in plants with antioxidant, anti-inflammatory, and immunomodulatory effects [40,41]. It has shown promising preventive and therapeutic effects in experimental models of chronic inflammatory and autoimmune diseases [42]. Chrysophanol, a major component of plant extracts used in many traditional Chinese medicines (TCMs), has shown diabetes-improving, anti-inflammatory, anti-cancer, neuroprotective, and liver-protecting effects [43]. Genistein is a kind of isoflavone mainly extracted from soybean, which has anti-inflammatory, antioxidant, and anti-cancer effects [44]. Orobol, a derivative of genistein, has the ability to improve acne inflammation caused by Propionibacterium acnes [45].

Gene ontology (GO) analysis showed that *Flemingia philippinensis* could improve inflammation through peptidyl-serine phosphorylation, peptidyl-serine modification, peptidyl-tyrosine phosphorylation, the response to the molecule of bacterial origin, and other biological processes. Phosphorylation is a widespread type of post-translational modification. More than 30% of proteins in cells can be phosphorylated to regulate intracellular signal transduction and metabolic processes. Therefore, phosphorylation is the most basic, universal, and important mechanism that regulates and controls protein activity and function [46]. Studies have shown that phosphorylation can regulate the activation of the inflammasome by targeting different inflammatory components, including NLRP3, NLRC4, IFI16, ASC, and Caspase-1. A large number of phosphorylation events can regulate the initiation, self-assembly, localization, and degradation of NLRP3 inflammatory cells [47]. Analysis of the cellular components revealed that the main targets of *Flemingia philippinensis* in improving inflammation are the membrane raft, membrane microdomain, membrane region, and apical part of the cell. Membrane microregions are relatively stable microregions on the membrane that are rich in specific proteins and lipids (including cholesterol, glycosphingolipids, sphingolipids, lipid rafts, and membrane caveolae). Related studies have found that cytokine stimulation during inflammation can induce Fcγ receptor I to accumulate in membrane microregions, thereby promoting the binding of immune complexes to monomeric IgG [48]. Molecular functions include nuclear receptor activity, ligand-activated transcription factor activity, protein tyrosine kinase activity, and serine/threonine protein kinase activity. Serine/threonine protein kinases have been shown to alleviate inflammation by phosphorylating serine and threonine residues on proteins. Receptor-interacting serine/threonine protein kinase 1(RIPK1) is a cytosolic protein kinase that can ameliorate inflammation [49].

Kyoto Encyclopedia of Genes and Genomes (KEGG) pathway analysis showed that the effect of *Flemingia philippinensis* on inflammation was mainly involved in EGFR tyrosine kinase inhibitor resistance, the PI3K–AKT signaling pathway, and the HIF-1 signaling pathway. EGFR is the receptor for epithelial growth factor (EGF) cell proliferation and signal transduction. PI3K–AKT is a classical intracellular signaling pathway, which is involved in the regulation of many physiological and pathological processes. Among these three inflammation-related pathways, the PI3K–Akt signaling pathway has the largest number of genes and thus may be a key pathway to improving inflammation. Relevant studies have shown that activating EGFR can activate the PI3K–AKT signaling pathway [50]. Activation of the PI3K–AKT signaling pathway plays a key role in acute and chronic inflammation [51,52]. At the same time, the PI3K–AKT signaling pathway can be involved in the regulation of multiple biological processes, such as cell proliferation, apoptosis, and glucose metabolism [53]. In addition, activated AKT can promote the expression and secretion of proinflammatory cytokines by activating the NF–κB pathway, leading to an imbalance of cytokine secretion and a series of inflammatory responses [54]. HIF-1 is a heterodimer composed of two subunits, Hif-1α and HIF-1β, whose transcriptional activity is triggered by hypoxia. It plays an important role in the development of the cardiovascular system, immune system, and cartilage [55]. Studies have found that activated HIF-1α can directly bind to the promoter of the TNF-α gene, thereby increasing the expression of TNF-α [56]. Therefore, the main mechanism by which *Flemingia philippinensis* ameliorated inflammation in this study may have included *Flemingia philippinensis* reducing cellular inflammation by regulating EGFR tyrosine kinase inhibitor resistance, the PI3K–AKT signaling pathway, and HIF-1 signaling pathway. These results indicated that multiple biological processes were involved in the anti-inflammatory effect of *Flemingia philippinensis*, and that the anti-inflammatory effect of *Flemingia philippinensis* was mediated by multiple components, targets, and pathways.

The results of this study provide strong theoretical support for improvements in inflammation to be achieved by *Flemingia philippinensis*. However, there are still some limitations in this study. Firstly, the information obtained from online databases is based on searched and predicted data; hence, unverified and undocumented compounds or targets may not be included in the analysis of this study. Secondly, the current research on the quantitative determination of the 29 compounds is not yet perfected; hence, future studies should focus on the quantification of these compounds. Thirdly, future research should investigate the effective components, absorption pathways, and metabolic forms of bioactive substances in *Flemingia philippinensis*. Fourthly, this study does not encompass polysaccharides, proteins, and other macromolecular compounds, which also require further investigation. Therefore, further research is required to explore the potential molecular mechanisms by which *Flemingia philippinensis* improves inflammation in vitro and in vivo.

## 5. Conclusions

In this study, network pharmacology and molecular docking methods were used to explore the mechanism by which *Flemingia philippinensis* ameliorated inflammation. The results showed that *Flemingia philippinensis* had the characteristics of multi-component, multi-target, and multi-pathway involvement in improving inflammation. However, while the results of this study, based on database analysis and network visualization, show the effect of key components on improving inflammation, they lack data on the effect of drug dosage. Therefore, these findings need further verification through cell and animal experiments in the future. However, the results of this study provide a theoretical basis for the development of multi-component, multi-target, and multi-pathway drugs for improving inflammation-related conditions. This study not only fills the research gap regarding the anti-inflammatory mechanisms of *Flemingia philippinensis* but also provides a reference method for the in-depth pharmacological study of other plants.

## Figures and Tables

**Table 1:** Information on the active ingredients of *Flemingia philippinensis*.

NO.	Molecule Name	Molecular Formula	Molecular Weight	GI Absorption	Number of Drug-like “YES”
1	(2S)-Liquiritigenin	C_15_H_13_O_4_	256.25	High	5
2	Monopalmitin	C_19_H_38_O_4_	330.50	High	3
3	2′-Hydroxygenistein	C_15_H_10_O_6_	286.24	High	5
4	Luteolin	C_15_H_10_O_6_	286.24	High	5
5	1,8-Cineole	C_10_H_18_O	154.28	High	3
6	Cubenol	C_15_H_26_O	222.37	High	4
7	Formononetin	C_16_H_12_O_4_	268.26	High	5
8	Orobol	C_15_H_10_O_6_	286.24	High	5
9	Chrysophanol	C_15_H_10_O_4_	254.24	High	5
10	Physcion	C_16_H_12_O_5_	284.26	High	5
11	Dihydrodaidzein	C_15_H_12_O_4_	256.25	High	5
12	4′,7-Dihydroxyflavanone	C_15_H_10_O_5_	254.24	High	5
13	Quercetin	C_15_H_10_O_7_	302.24	High	5
14	3′-O-Methylorobol	C_16_H_12_O_6_	300.26	High	5
15	Elemene	C_15_H_24_	204.39	High	4
16	Diisobutyl phthalate	C_16_H_22_O_4_	278.34	High	5
17	o-Thymol	C_10_H_14_O	150.24	High	3
18	Flemiphilippinin D	C_25_H_28_O_6_	424.50	High	4
19	Flemiphilippinin C	C_26_H_26_O_6_	406.46	High	4
21	Medicagol	C_16_H_8_O_6_	296.23	High	5
21	Wedelolactone	C_16_H_10_O_7_	314.25	High	5
22	Flemichin D	C_25_H_26_O_6_	422.47	High	4
23	Genistein	C_15_H_10_O_5_	270.24	High	5
24	Kaempferol	C_15_H_10_O_6_	286.24	High	5
25	Lupinifolin	C_25_H_26_O_5_	406.50	High	4
26	Prunetin	C_16_H_12_O_5_	284.26	High	5
27	Naringenin	C_15_H_12_O_5_	272.25	High	5
28	Lupeol	C_30_H_50_O	426.80	Low	3
29	Palmitic acid	C_16_H_32_O_2_	256.42	High	3

**Table 2:** Top 10 key core targets.

NO.	Gene Name	Target Protein	Degree	Betweenness Centrality	Closeness Centrality
1	AKT1	Serine/threonine-protein kinase AKT	49	0.041 266	0.962 264
2	TNF	TNF-alpha	47	0.030 942	0.927 273
3	BCL2	Apoptosis regulator Bcl-2	47	0.028 528	0.927 273
4	ALB	Serum albumin	47	0.034 065	0.927 273
5	ESR1	Estrogen receptor alpha	46	0.031 392	0.910 714
6	PPARG	Peroxisome proliferator-activated receptor gamma	44	0.026 722	0.879 310
7	EGFR	Epidermal growth factor receptor erbB1	44	0.021 417	0.879 310
8	PTGS2	Cyclooxygenase-2	42	0.018 727	0.850 000
9	SRC	Tyrosine-protein kinase SRC	41	0.014 351	0.836 066
10	HSP90AA1	Heat shock protein HSP 90-alpha	40	0.016 795	0.822 581

**Table 3:** Molecular docking binding energies of key targets and components (kcal/mol).

Key Molecular Components	Core Targets					Average Binding Energy
	AKT1	TNF	BCL2	ALB	ESR1	
Flemichin D	−9.57	−8.70	−8.32	−8.20	−7.54	−8.47
Naringenin	−7.82	−7.48	−7.01	−7.60	−7.96	−7.57
Chrysophanol	−8.00	−7.20	−6.25	−8.85	−7.29	−7.52
Genistein	−7.60	−6.67	−5.78	−6.74	−8.03	−6.96
Orobol	−7.25	−6.76	−6.23	−7.02	−8.10	−7.07

## Data Availability

The original contributions presented in this study are included in the article’s material. Further inquiries can be directed to the corresponding author.
